# The Management of Motor Neuropathy With Plasmapheresis in a Patient With Acute Porphyria: A Case Report

**DOI:** 10.7759/cureus.43196

**Published:** 2023-08-09

**Authors:** Umar Maqbool, Muhammad Ahmad Khan, Abdullah Maqbool, Wajeeha Aslam, Muhammad Arsal Naseem

**Affiliations:** 1 Internal Medicine, Mayo Hospital, Lahore, PAK; 2 Internal Medicine, Arif Memorial Teaching Hospital, Lahore, PAK

**Keywords:** acute abdomen, case report, plasmapheresis, motor neuropathy, acute porphyria

## Abstract

Acute porphyria results from a deficiency of enzymes crucial for the heme synthesis process. This deficiency leads to elevated levels of intermediates, resulting in the characteristic symptoms of porphyrias such as abdominal and limb pain, neuropsychiatric issues, and sensitivity to light. In this report, we present the case of a 15-year-old male who experienced deteriorating motor neuropathy and recurrent bouts of abdominal pain. Numerous investigations were conducted, eventually leading to a diagnosis of acute porphyria. Despite attempts with hemin and glucose therapy, his motor neuropathy did not improve. However, significant progress was observed following plasmapheresis sessions. This case emphasizes the importance of considering acute porphyrias as a potential cause when managing patients with acute abdominal problems. By fostering a collaborative approach involving hematologists, physicians, neurologists, and surgeons, timely diagnosis and effective management of this condition can be achieved.

## Introduction

Porphyrins are cyclical compounds containing tetrapyrrole rings, which readily bind metal ions. Heme is a metalloporphyrin that constitutes hemoglobin, myoglobin, cytochrome oxidase system, and catalase. Porphyrin synthesis predominantly occurs in the liver and the bone marrow. Heme synthesis involves a series of reactions of cytosolic and mitochondrial enzymes. Acquired or inherited defects in these enzymes lead to defects in heme synthesis, resulting in the accumulation of intermediates of the synthetic pathway. These defects are called porphyrias.

Porphyrias classically present with red-colored urine, photosensitivity, and abdominal and neuropsychiatric symptoms [1]. The enzyme defects before the synthesis of the tetrapyrrole ring predominantly lead to abdominal and neurological symptoms. The enzyme defects after the synthesis of the tetrapyrrole ring lead to phototoxicity. Porphyrias are classified into hepatic and erythropoietic forms. Hepatic porphyrias are further classified into acute and chronic types. Acute porphyrias are typically diagnosed by measuring urinary porphobilinogen levels, which are elevated. Enzyme studies can be undertaken to determine the particular enzyme defect. Blood tests typically show microcytic anemia and hyponatremia [2].

A characteristic history of acute attacks constituting abdominal pain, vomiting, constipation, skin lesions, and mixed neuropathy raises the suspicion of porphyrias [3]. Acute attacks of porphyria are managed by relieving the patient’s abdominal pain and vomiting. Intravenous administration of glucose and heme reduces the synthesis of porphyria intermediates, resulting in the improvement of the patient’s symptoms over longer durations. Sunlight exposure should be limited to reduce photosensitivity. The main long-term outcome of porphyrias is chronic neuropathy. This article demonstrates that plasmapheresis can be an effective management option for porphyria neuropathy.

## Case presentation

A 15-year-old male presented to the medical outpatient department of Mayo Hospital Lahore on July 7, 2022, with complaints of difficulty walking for one year. He had been in his usual state of health around one year ago when he developed constipation, sudden in onset, associated with diffuse abdominal pain which had been relieved by enemas and laxatives given at a local hospital. An abdominal ultrasound scan had been performed at that time, which had been normal. Two days later, he started experiencing weakness in his legs, which he described as an inability to put weight on his feet. The weakness had been gradual, and progressive, initially making it difficult for him to walk but had progressed to involve upper limbs over the next three days. It had been associated with numbness of forearms and legs (up to knee joints). The weakness had progressed to make him bedridden within two months. At that time, the patient had started to notice that he had been passing red-colored urine. However, he had not experienced any urinary retention, urinary incontinence, or fecal incontinence. He had started experiencing generalized tonic-clonic fits eight months back. He had multiple episodes of fits over the last eight months. The fits usually lasted two to three minutes. These episodes had been associated with low-grade fever. He had given a history of intermittent abdominal pain and constipation for the last eight months, which had been relieved by laxatives. He had not experienced bloating, weight loss, or mucous in stool. He only had generalized non-specific abdominal pain. The patient had also started experiencing hair fall, cold intolerance, and constipation at that time. There had been no history of oral ulcers, photophobia, skin rash, blurry vision, or diplopia. There had also been no history of headache, scalp tenderness, jaw claudication, hemoptysis, shortness of breath, drug abuse, and weight loss. His parents had noted his aggressive behavior since the beginning of his symptoms. He had started to get angry with his family members over minor things. However, there had been no history of inappropriate laughs, weeping spells, suicidal ideation, and hallucinations. The patient has a brother who has no such complaints. His family history was also insignificant. The patient had a good nutritional intake.

**Figure 1:**
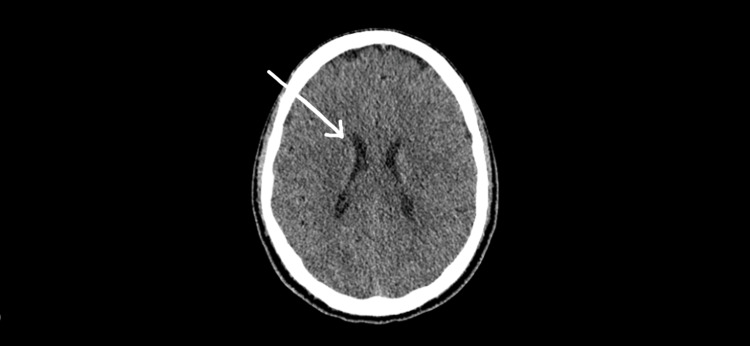
Axial CT brain showing a very mild ventricular dilatation, straight midline, and no pathology CT: computed tomography

On general physical examination, the patient's hands and conjunctiva were pale. He had marked muscle wasting visibly on his arms and calves. His CNS examination showed intact cranial nerves and cerebellar function. He had impaired pin-prick sensation up to the knee joint and mid-forearm. He had decreased bulk and tone in bilateral upper and lower limbs. There were no visible fasciculations. Power in proximal bilateral upper limbs was 5/5 and distal bilateral upper limbs was 2/5. Power in proximal bilateral lower limbs was 4/5 and distal bilateral lower limbs was 0/5. The patient had bilateral wrist and foot drops. His abdominal examination showed a soft and non-tender abdomen. There were no visible lesions, no palpable masses, and no distention. Auscultation showed normal bowel sounds. His digital rectal examination was also normal. The patient had his CT and MRI brain done at a private hospital before his presentation to the Mayo Hospital Lahore. His CT brain showed a very mild ventricular dilatation. There were no abnormalities on the CT scan (Figure 1).

**Figure 2:**
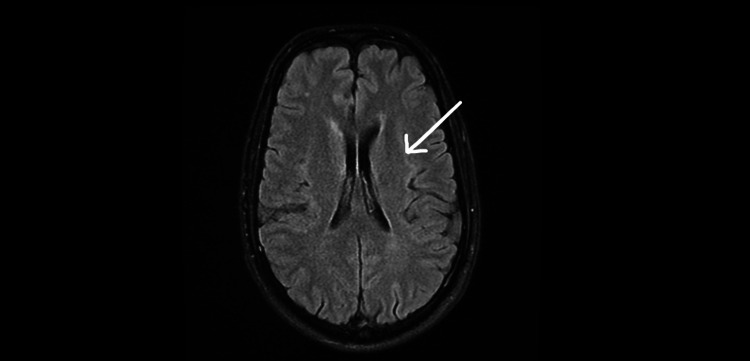
Axial MRI Brain showing a clear distinction between white and gray matter and no pathology MRI: magnetic resonance imaging

His MRI was also completely normal and no pathology was identified (Figure 2).

The patient was admitted to the medical ward. His complete blood count and peripheral smear showed hypochromic, microcytic red blood cells with a decreased reticulocyte count (Hb: 8.7 g/dL, MCV: 67 μm3, reticulocytes: 1.4%). The patient was scheduled for nerve conduction studies to determine the type of neuropathy responsible for distal muscle weakness. His nerve conduction studies suggested mixed sensory and motor polyneuropathy more severe distally and in the lower limbs. His electroencephalography (EEG) showed a mixture of theta and delta waves with no epileptic waveforms. The patient’s CSF was drawn through a lumbar puncture and sent for CSF analysis to rule out acute and chronic inflammatory polyneuropathies. His CSF was negative for xanthochromia, atypical cells, and microorganisms with a few RBCs and lymphocytes in the background.

**Figure 3:**
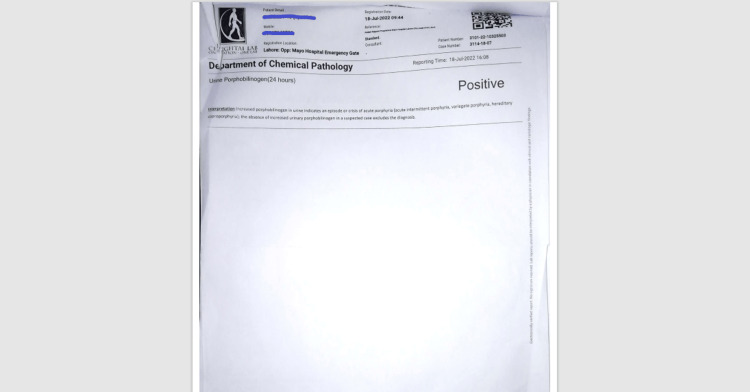
24-hour urine report showing positive urinary porphobilinogen

The patient had two acute attacks of abdominal pain and fits during his stay in the ward. His abdominal ultrasound scans were normal during both attacks. He was given antispasmodics, which improved his pain. His labs during the acute attack consistently showed hyponatremia (Na+: 119 mmol/L). While all other neuropathy causes were excluded, the patient’s hyponatremia and red-colored urine raised the suspicion that he was suffering from acute porphyria. His 24-hour urine examination showed increased urinary porphyrins (Figure 3).

His ESR during the acute attack was also elevated at 34 mm/hour (normal: less than 10 mm/hour). These investigations confirmed that the patient was suffering from acute porphyria, which was responsible for his abdominal and neuropsychiatric symptoms. His lead levels were ordered to exclude lead poisoning, which came out to be normal (Pb: 0.5 µg/dL).

The patient was first started on intravenous 5% dextrose saline for nine days. However, he failed to show any response to this therapy. He was then started on intravenous hemin therapy at a dose of 4 mg/kg of body weight. Intravenous heme administration resulted in an improvement in his abdominal symptoms and acute attacks but showed no improvement in motor neuropathy. It was finally decided to perform plasmapheresis to improve his motor symptoms. The patient underwent five sessions of plasmapheresis on alternate days. After plasmapheresis, his power in bilateral lower limbs improved to 3/5. He also showed improvement in his psychiatric symptoms. The patient did not experience fits after this therapy. He was discharged from the medical ward and referred to a physiotherapist. He was advised to follow up in the outpatient department. He did not develop any complications during his follow-up visits.

## Discussion

Heme synthesis involves several enzyme reactions in the cytosol and the mitochondria that work in a sequential pathway, ultimately leading to heme formation. Deficiency of these enzymes results in porphyrias. A typical clinical characteristic to determine the level of enzymatic defect is the presence of phototoxicity. If there is no phototoxicity, the enzymatic defect is likely before the synthesis of the tetrapyrrole ring. In this case, the patient had recurrent attacks of abdominal pain and neuropsychiatric symptoms. However, there was no history of phototoxicity. Although enzyme studies to determine the exact enzymatic defect could not be performed due to financial reasons, the defect most likely involves the aminolevulinic acid (ALA) dehydratase or porphobilinogen deaminase enzymes. Regardless, this case report focuses on the management of neuropsychiatric symptoms encountered in acute porphyrias. Neuropathic pain is most likely responsible for the recurrent attacks of abdominal pain [4]. It is postulated that ALA is a neurotoxic compound that is responsible for progressive neurological impairment [5]. The progressive nature of neurological symptoms, as seen in this case, can ultimately lead to respiratory insufficiency requiring mechanical ventilation [6]. In recurrent attacks, muscle weakness can precede abdominal symptoms [6].

Acute attacks of neuropathic pain can be managed with hemin infusions and opioids [7]. Although the clinical symptoms of the patient, including abdominal pain and psychiatric features, usually show an improvement with this treatment, nerve conduction studies show that axonal neuropathy takes longer to recover. In most cases, the recovery is not usually complete [8]. Chronic neuropathic pain can be managed with gabapentinoids [9]. This patient was initially treated with dextrose and hemin administration. These treatment modalities failed to improve his neuropsychiatric symptoms. Ultimately, five sessions of plasmapheresis significantly improved his symptoms. There is a case report of a 56-year-old woman who presented with motor neuropathy and supranuclear ophthalmoplegia secondary to acute porphyria [10]. Her symptoms gradually improved during plasmapheresis sessions. There is also documented evidence that the levels of peptides increase during acute attacks of porphyrias [11]. Combination therapy of somatostatin and plasmapheresis has proven to be beneficial in these cases [12]. Plasmapheresis certainly plays an important role in the management of chronic neuropathy of acute porphyrias. The underlying mechanism of the effectiveness of plasmapheresis lies in the removal of neurotoxic porphyrin intermediates from the serum. This case highlights the importance of plasmapheresis in the management of chronic polyneuropathy of porphyrias. Further research is required in this regard. This case provides a significant contribution to that end.

## Conclusions

Acute porphyrias present with features of severe abdominal and limb pain that is neuropathic. The diagnosis is usually delayed as patients with abdominal pain are typically referred to the surgical team. It is important to keep porphyria in the differential diagnosis in cases where no cause of abdominal pain can be identified and the CT abdomen is normal. Lead poisoning should be excluded as plumbism can lead to porphyria symptoms. While dextrose and hemin therapy does have a significant role in managing acute porphyria, plasmapheresis has demonstrated significant benefits in patients suffering from chronic motor neuropathy. Though this case demonstrates successful management of porphyria neuropathy with plasmapheresis, further research is required to gain deeper insights and gather better evidence. This case report highlights the need for further research into this aspect to explore the long-term outcomes of plasmapheresis in porphyria.
